# Lipid droplets as ubiquitous fat storage organelles in *C. elegans*

**DOI:** 10.1186/1471-2121-11-96

**Published:** 2010-12-08

**Authors:** Shaobing O Zhang, Rhonda Trimble, Fengli Guo, Ho Yi Mak

**Affiliations:** 1 Stowers Institute for Medical Research, 1000 E. 50th Street, Kansas City, MO 64110, USA; 2 Deartment of Molecular and Integrative Physiology, University of Kansas Medical Center, Kansas City, KS 66160, USA

## Abstract

**Background:**

Lipid droplets are a class of eukaryotic cell organelles for storage of neutral fat such as triacylglycerol (TAG) and cholesterol ester (CE). We and others have recently reported that lysosome-related organelles (LROs) are not fat storage structures in the nematode *C. elegans*. We also reported the formation of enlarged lipid droplets in a class of peroxisomal fatty acid β-oxidation mutants. In the present study, we seek to provide further evidence on the organelle nature and biophysical properties of fat storage structures in wild-type and mutant *C. elegans*.

**Results:**

In this study, we provide biochemical, histological and ultrastructural evidence of lipid droplets in wild-type and mutant *C. elegans *that lack lysosome related organelles (LROs). The formation of lipid droplets and the targeting of BODIPY fatty acid analogs to lipid droplets in live animals are not dependent on lysosomal trafficking or peroxisome dysfunction. However, the targeting of Nile Red to lipid droplets in live animals occurs only in mutants with defective peroxisomes. Nile Red labelled-lipid droplets are characterized by a fluorescence emission spectrum distinct from that of Nile Red labelled-LROs. Moreover, we show that the recently developed post-fix Nile Red staining method labels lipid droplets exclusively.

**Conclusions:**

Our results demonstrate lipid droplets as ubiquitous fat storage organelles and provide a unified explanation for previous studies on fat labelling methods in *C. elegans*. These results have important applications to the studies of fat storage and lipid droplet regulation in the powerful genetic system, *C. elegans*.

## Background

Lipid droplets are defined as a class of organelles for storing neutral fat such as triacylglycerol (TAG) and cholesterol ester (CE) in eukaryotes [1,2]. Lipid droplets are spherical structures delimited by a phospholipid monolayer [3] that is coated by various proteins including Adipophilin, Perilipin, and adipose triglyceride lipase (ATGL) [4-6]. The size and content of lipid droplets can be dynamically regulated by both metabolic pathways and coat proteins. Studies of how lipid droplets are regulated will undoubtedly yield key insights into the understanding of obesity, diabetes, and other metabolic diseases [1,2].

The nematode *C. elegans *has emerged as an important model to study fat metabolism. In *C. elegans*, the majority of fat is stored in gut epithelial cells. However, the organelle nature and biophysical properties of fat storage structures are not fully defined. The putative fat storage structures have been given different names such as gut granules or lysosome-related organelles (LROs) [7], vesicles distinct from lysosome-related organelles [8], and lipid droplets [9-11]. These names reflect the different approaches to and current insufficient understanding of *C. elegans *fat storage structures. Vital labelling with Nile Red or a BODIPY fatty acid analog (BODIPY in abbreviation) was introduced as a proxy for qualitative and quantitative measurement of fat in *C. elegans *[12]. Vital Nile Red and vital BODIPY co-label a population of structures in gut epithelial cells, except that BODIPY but not Nile Red weakly labels extra structures in gut epithelial cells and strongly labels structures in hypodermal cells [13]. Because vital staining is conducive to screening and live imaging, it has been widely used to screen for fat storage mutants and to measure fat levels in *C. elegans *[14-18]. However, vital Nile Red-labelled structures were recently shown to be LROs in the study of a class of *glo *mutants [7]. In the *glo *mutants, Nile Red staining and LROs were lost. However, quantitative TAG measurement by gas chromatography-mass spectrometry (GC-MS) revealed that fat levels were unaltered [19]. Moreover, a recent study also suggested that Nile Red-labelled structures and the majority of BODIPY-labelled structures were LROs [8]. This recent study and another study [20] demonstrated that vital Nile Red and vital BODIPY staining intensities did not necessarily correlate with fat levels measured by GC-MS in mutants previously studied. Instead, post-fix Oil-Red-O [8] and post-fix Nile Red [20] staining intensities correlated more closely with biochemically verified fat levels. The underlying principles of the two recent staining approaches are unknown. But they both relied on fixation of animals.

In a previous report, we showed lipid droplet expansion in a class of peroxisomal fatty acid β-oxidation mutants: *maoc-1*, *dhs-28*, and *daf-22 *[21]. MAOC-1/hydratase, DHS-28/dehydrogenase, and DAF-22/thiolase carry out three successive reactions in the peroxisomal fatty acid β-oxidation pathway. Here, we report that 1) wild-type *C. elegans *has lipid droplets that display the same fluorescence, density, and ultrastructural properties as enlarged lipid droplets in peroxisomal β-oxidation mutants. 2) Lipid droplets in wild-type animals are vital-labelled weakly by BODIPY but not by Nile Red, while LROs are vital-labelled strongly by both. 3) Lipid droplets in peroxisomal β-oxidation mutants can be vital-labelled by Nile Red. 4) Nile Red-labelled lipid droplets can be distinguished from LROs by a distinct fluorescence emission spectrum. 5) The post-fix Nile Red staining approach labels lipid droplets solely. These results demonstrate the complexity of lipophilic dye trafficking in gut epithelial cells and should lay down a foundation for future studies of lipid droplets in *C. elegans*.

## Results

### Both LROs and lipid droplets can be vital-labelled by BODIPY fatty acid analogs

**Figure 1:**
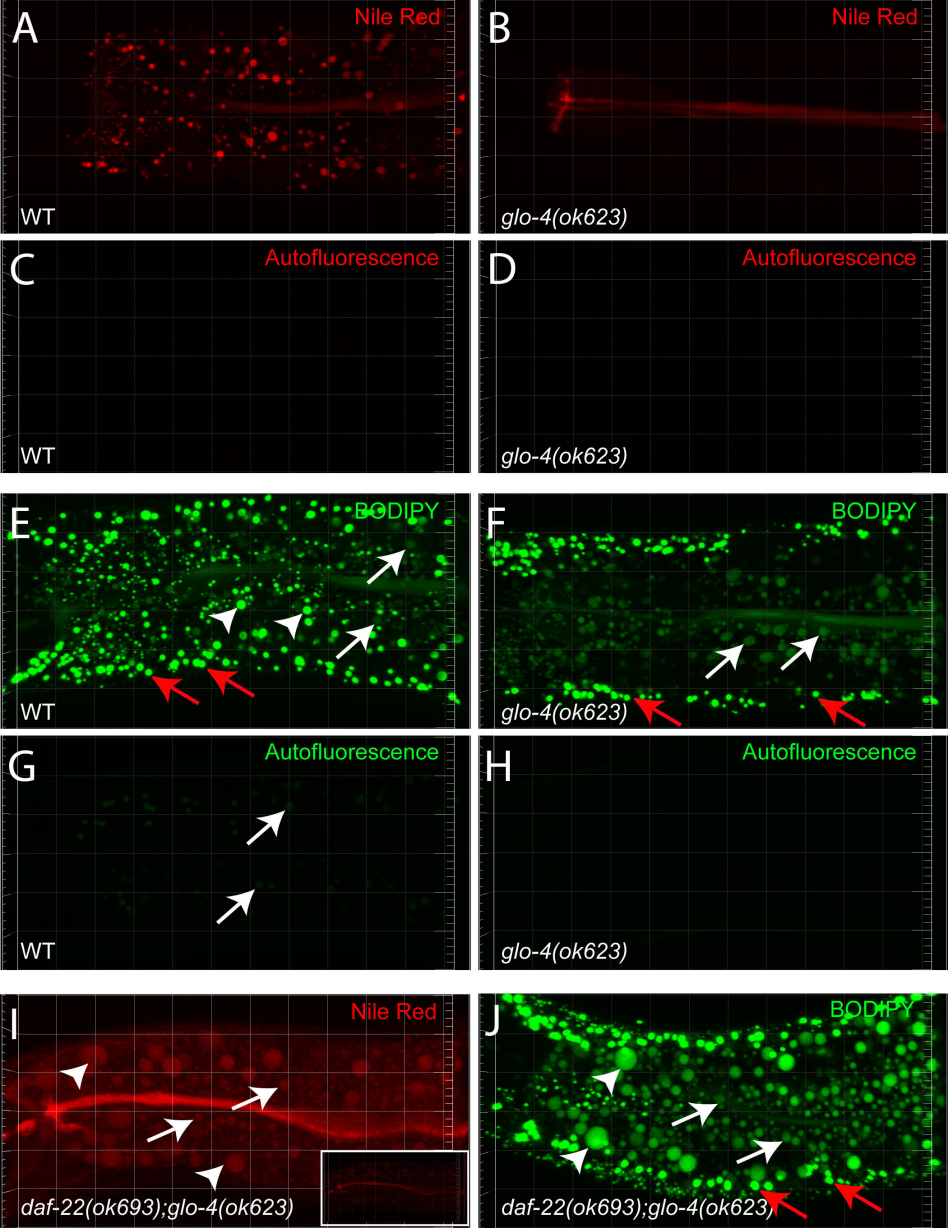
**Different labelling properties of Nile Red and BODIPY in live animals**. (A and B) Nile Red labelled intracellular structures in gut epithelial cells in wild-type (WT) animals but not in *glo-4 *mutants. Stained midlines were gut lumen (the same in other images if not otherwise indicated). (C and D) No autofluorescence in unstained wild-type or *glo-4 *animals was detected under the same Nile Red imaging condition. (E) BODIPY labelled a population of structures with high intensity (arrowheads) and labelled another population of structures with low intensity (white arrows) in gut epithelia cells in wild-type animals. (F) In *glo-4 *animals, only the low-intensity BODIPY structures remained in gut epithelial cells (arrows). Red arrows point to structures in hypodermal cells (E, F and J). (G and H) LRO-specific autofluorescence (arrows) was detected under the same BODIPY imaging condition in unstained wild-type animals but not in unstained *glo-4 *animals. (I and J) Nile Red and BODIPY labelled both enlarged lipid droplets (arrowheads) and putative small lipid droplets (white arrows) in live *daf-22; glo-4 *mutants. To better visualize Nile Red-stained structures, brightness of the original image (I, inset) was enhanced. All animals were late stage L4 if not other otherwise indicated. All images were 3-D projections of 9 μm confocal stacks. Gridlines, 10 μm.

To investigate whether vital staining by Nile Red or BODIPY could label both LROs and lipid droplets, we grew wild-type and *glo-4(ok623) *animals on OP50 *E. coli *diet supplemented with Nile Red or BODIPY. *glo-4 *encodes a putative guanine nucleotide exchange factor (GEF) for the GLO-1 Rab GTPase. *glo-1 *and *glo-4 *mutants lacked LROs [7]. Consistent with a previous report [7], there was no detectable Nile Red-labelled structure in *glo-4 *mutants (Figure 1A and 1B). Using the same imaging parameters, no autofluorescence was detected in either wild-type or *glo-4 *animals that were not vital-stained with Nile Red (Figure 1C and 1D). These results support the idea that Nile Red labels LROs only. In contrast, we observed in gut epithelial cells of wild-type animals two populations of BODIPY-labelled structures, one with high fluorescence intensity and the other with lower fluorescence intensity (Figure 1E). In *glo-4 *mutants, the low-intensity population remained (Figure 1F), while the high-intensity population (Figure 1E) and LRO-specific autofluorescence [7] were lost (Figure 1G and 1H). These results suggest that the high-intensity BODIPY structures are LROs while the low-intensity BODIPY structures are putative fat storage structures.

To investigate whether the high-intensity BODIPY structures are LROs, we grew wild-type animals bearing a transgenic marker for LROs (*hjIs9 [glo-1::gfp]*) on OP50 diet supplemented with a red BODIPY fatty acid analog, BODIPY558/568-C12. Indeed, GLO-1::GFP encircled high-intensity red BODIPY structures (Additional File 1). This is consistent with a previous finding that the high-intensity green BODIPY signal co-localizes with lysoTracker, a marker for LROs [8]. However, red BODIPY also labelled GLO-1::GFP-negative structures, albeit with lower fluorescence intensity (Additional File 1). In peroxisomal β-oxidation mutants, enlarged lipid droplets which are GLO-1::GFP-negative, can be labelled by BODIPY [21]. Thus, the GLO-1::GFP-negative structures with low BODIPY intensity in wild-type animals may also be genuine fat storage structures, i.e., putative lipid droplets.

**Figure 2:**
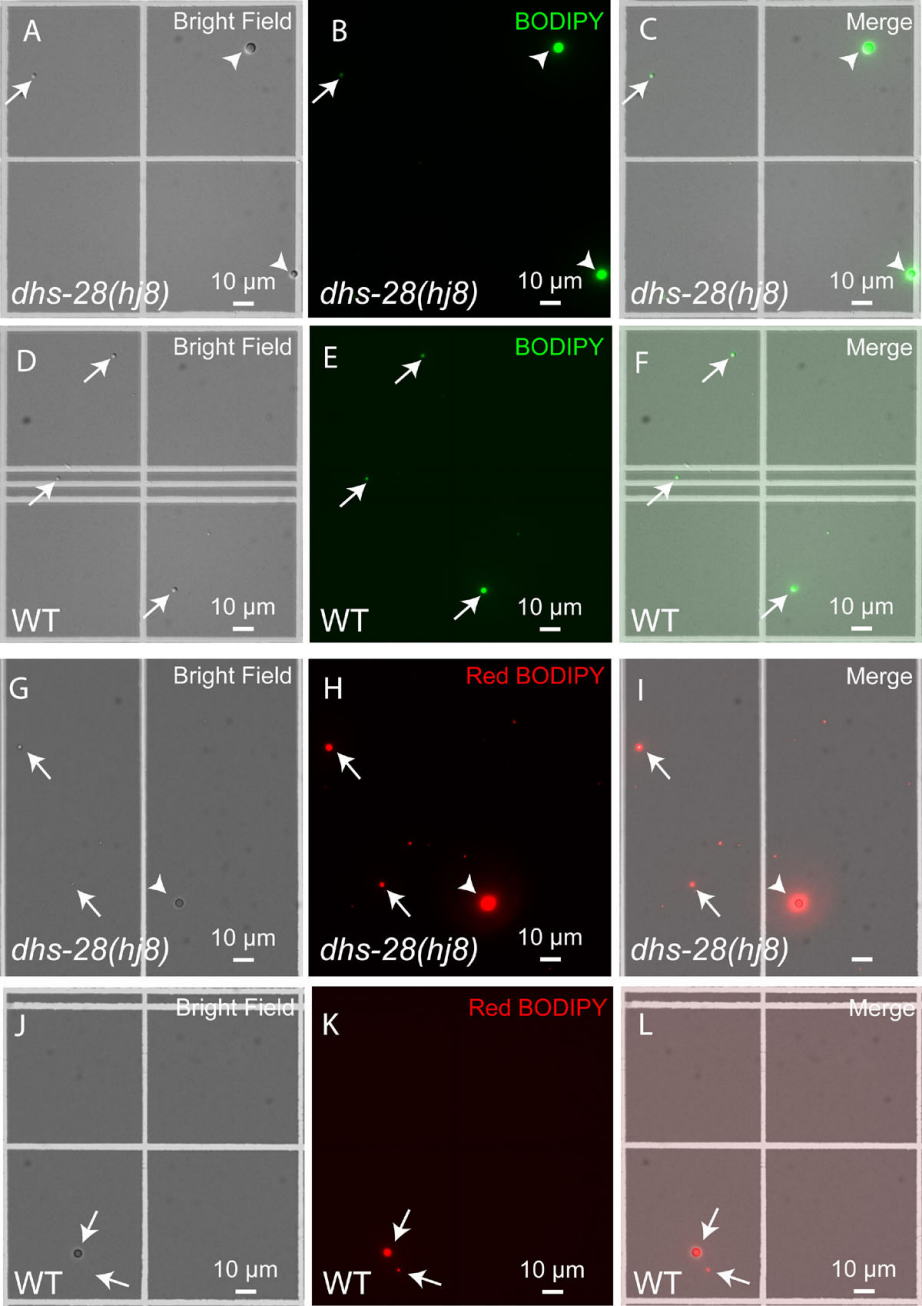
**Isolation of lipid droplets from vital BODIPY-labelled animals**. (A-C) Green BODIPY-labelled enlarged (arrowheads) and small (arrows) lipid droplets were isolated from *dhs-28 *mutants. (D-F) Green BODIPY-labelled small lipid droplets (arrows) were isolated from wild-type animals. Red BODIPY-labelled lipid droplets were isolated from *dhs-28 *mutants (G-I) and from wild-type animals (J-L). Images were taken on an inverted wide-field microscope.

To confirm that bona fide neutral fat storage structures in live wild-type animals can be labelled by BODIPY, we adopted and modified previous differential centrifugation approaches [22,23] to partially isolate lipid droplets from *dhs-28(hj8) *and wild-type animals that were vital-labelled by BODIPY. This approach exploits the fact that lipid droplets have a lower density than water and most other intracellular organelles. Indeed, enlarged and small lipid droplets isolated from *dhs-28 *mutants were shown to be labelled by green BODIPY (Figure 2A-2C). Also, small putative lipid droplets isolated from wild-type animals were shown to be labelled by green BODIPY, albeit with low intensity (Figure 2D-2F). Similarly, enlarged lipid droplets isolated from *dhs-28 *were labelled by red BODIPY(Figure 2G-2I), and small putative lipid droplets isolated from wild-type animals were labelled by red BODIPY with low intensity (Figure 2J-2L). Furthermore, we prepared lipid droplet fraction and cytoplasmic fraction from *dhs-28 *mutant animals bearing the *glo-1::gfp *transgene (*dhs-28;glo-1::gfp*). Western blot experiments showed that the lipid droplet fraction had no detectable GLO-1::GFP protein, which was found predominantly in the cytoplasm fraction (Additional File 2). This result demonstrated that our biochemical approach could yield lipid droplet fractions devoid of LROs.

### In peroxisomal β-oxidation mutants, lipid droplets can be vital-labelled by Nile Red and be distinguished from LROs by a distinct fluorescence spectrum

A previous report [24] and our unpublished observations (see below) demonstrated that Nile Red could vital-label enlarged lipid droplets and other small spherical structures in peroxisomal β-oxidation mutants. We have shown that although the loss of *glo-4 *function leads to a loss of LROs, it did not prevent the formation of enlarged lipid droplets in mutants with defective peroxisomes [21]. Interestingly, in *daf-22*; *glo-4 *double mutants, enlarged lipid droplets and small putative lipid droplets could be vital-labelled by Nile Red (Figure 1I) as well as by BODIPY (Figure 1J). It further supports the notion that, in contrast to lipid droplets in wild-type animals, lipid droplets in peroxisomal β-oxidation mutants can be vital-labelled by Nile Red.

**Figure 3:**
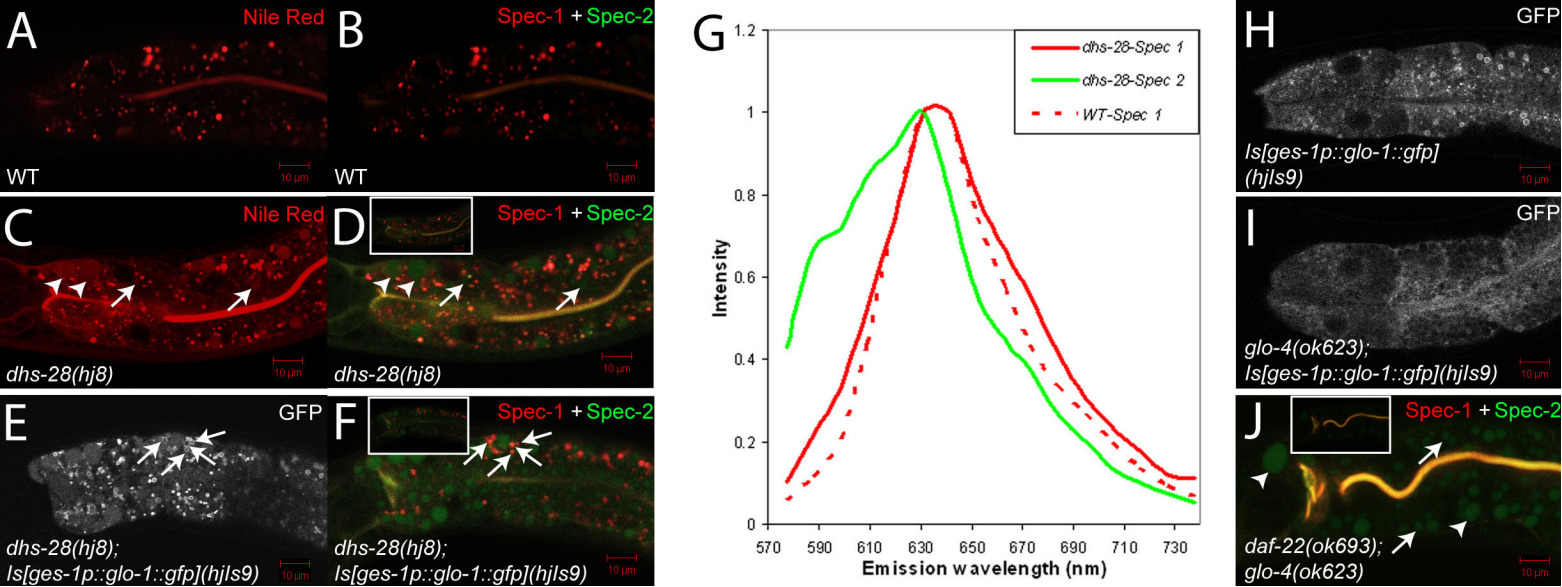
**Different fluorescence emission spectra of Nile Red-labelled LROs and lipid droplets**. (A) Nile Red labelled structures in live wild-type animals as revealed by channel mode confocal images. (B) These labelled structures (pseudo-colored in red) displayed the same emission spectrum (Spec-1) as revealed by linearly un-mixing lambda mode confocal images. (C) Nile Red labelled structures in live *dhs-28 *mutants as revealed by channel mode confocal images. (D) Nile Red labelled both Spec-1 structures (pseudo-colored in red) and Spec-2 structures (pseudo-colored in green) in live *dhs-28 *mutants as revealed by linearly un-mixing lambda mode confocal images. Spec-2s included enlarged lipid droplets (arrowheads) and putative small lipid droplets (arrows). To aid visualization of Spec-2 structures, brightness of the original un-mixed image (inset) was enhanced. The same was applied to F and J. (E and F) Spec-1 structures were mostly marked by the LRO marker GLO-1::GFP (arrows). (G) Emission spectrum profiles of Spec-1s in wild-type and *dhs-28 *animals and Spec-2s in *dhs-28 *animals. Intensity values were normalized. (H and I) LRO-specific GLO-1::GFP structures were lost in *glo-4 *mutants. (J) In *daf-22; glo-4 *mutants, LRO-specific Spec-1s were lost while enlarged (arrowheads) and small (arrows) lipid droplets-specific Spec-2s remained. All images were single confocal slices. Scale bars, 10 μm.

Here, we sought to compare fluorescence properties of Nile Red-labelled LROs in wild-type animals and Nile Red-labelled lipid droplets and LROs in peroxisomal β-oxidation mutants. We scanned Nile Red emission spectra in wild-type and *dhs-28 *animals. In wild-type animals, all Nile Red-positive structures, i.e., LROs, displayed the same emission spectrum that peaked at 641 nm (Figure 3A, 3B and 3G). We classified these structures as "Spec-1". In contrast, in *dhs-28 *mutants, there were structures with a different spectrum that peaked at 631 nm and shouldered at 590 nm and 610 nm in addition to Spec-1s (Figure 3C, 3D and 3G). We classified these structures as "Spec-2". In wild-type and *dhs-28 *mutant animals, the Spec-1s were mostly smaller than 3 μm in diameter and encircled by the LRO marker GLO-1::GFP (Figure 3E and 3F). In *dhs-28 *mutant animals, the Spec-2s were rather heterogeneous in size, ranging from below 1 μm to 8 μm in diameter (Figure 3D and 3F). Importantly, all enlarged lipid droplets were Spec-2s (Figure 3D and 3F). Consistent with the notion that *glo-4 *encodes a guanine nucleotide exchange factor for GLO-1, GLO-1::GFP marking was lost in *glo-4 *mutants (Figure 3H and 3I). In *daf-22*;*glo-4 *double mutants, all Spec-1s were lost, while Spec-2s, i.e., enlarged lipid droplets and small putative lipid droplets, remained (Figure 3J).

We next sought to obtain vital Nile Red-stained subcellular structures using our differential centrifugation procedure. To this end, we grew *dhs-28 *mutants on 10-cm NGM/OP50 plates that were overlaid with Nile Red. We then prepared lipid droplet fractions from ~12,000 *dhs-28 *animals harvested from 10-cm NGM/OP50/Nile Red plates, and pelleted cytoplasmic membranous organelles that included LROs. As shown in Additional File 3, isolated membrane pellets displayed Spec-1 spectrum similar to Spec-1 in intact *dhs-28 *animals; and isolated lipid droplets displayed Spec-2 spectrum similar to Spec-2 in intact *dhs-28 *animals. For reasons currently unknown, we noted that *dhs-28 *animals grown and stained on 10-cm NGM/OP50/Nile Red plates displayed more prominent 590 nm and 610 nm peaks of Spec-2s than their counterparts that were grown and stained on 6-cm NGM/ OP50/Nile Red plates (compare Additional File 3D with Figure 3G).

We also probed whether red BODIPY had different fluorescence emission spectra when labelling LROs vs. lipid droplets. In *dhs-28 *mutant animals bearing GLO-1::GFP marker, both GFP-encircled LROs and non-GFP-encircled large lipid droplets displayed the same emission spectrum peaked at 577 nm and shouldered at 620-631 nm (Additional File 4). Thus, BODIPY labelling did not distinguish lipid droplets from LROs as Nile Red labelling did.

We interpret these results as indicating that in wild-type *C. elegans*, lysosome related organelles (LROs) are vital-labelled by both BODIPY and Nile Red with high intensity. On the other hand, lipid droplets, the fat storage structures, can be vital-labelled by BODIPY with low intensity but not by Nile Red. Under special conditions such as peroxisomal β-oxidation dysfunction, lipid droplets can be labelled by Nile Red and be distinguished from LROs by a distinct fluorescence emission spectrum. Since in any situation, both BODIPY and Nile Red stain LROs strongly, fluorescence intensities of the two vital lipophilic dyes should be used with great caution as surrogates of intracellular fat levels.

### Post-fix Nile Red staining labels lipid droplets solely

Recently, it was found that the intensities of post-fix Nile Red or Oil-Red-O staining correlated more closely with quantitative TAG measurements by GC-MS than the intensities of vital Nile Red or BODIPY staining [8,20]. However, the underlying principle of the post-fix staining as a better proxy of fat level is not clear. The post-fix approach involves permeating cell membrane and intracellular membrane with isopropanol and freeze-fracture. This treatment presumably allows direct penetration of the two dyes.

**Figure 4:**
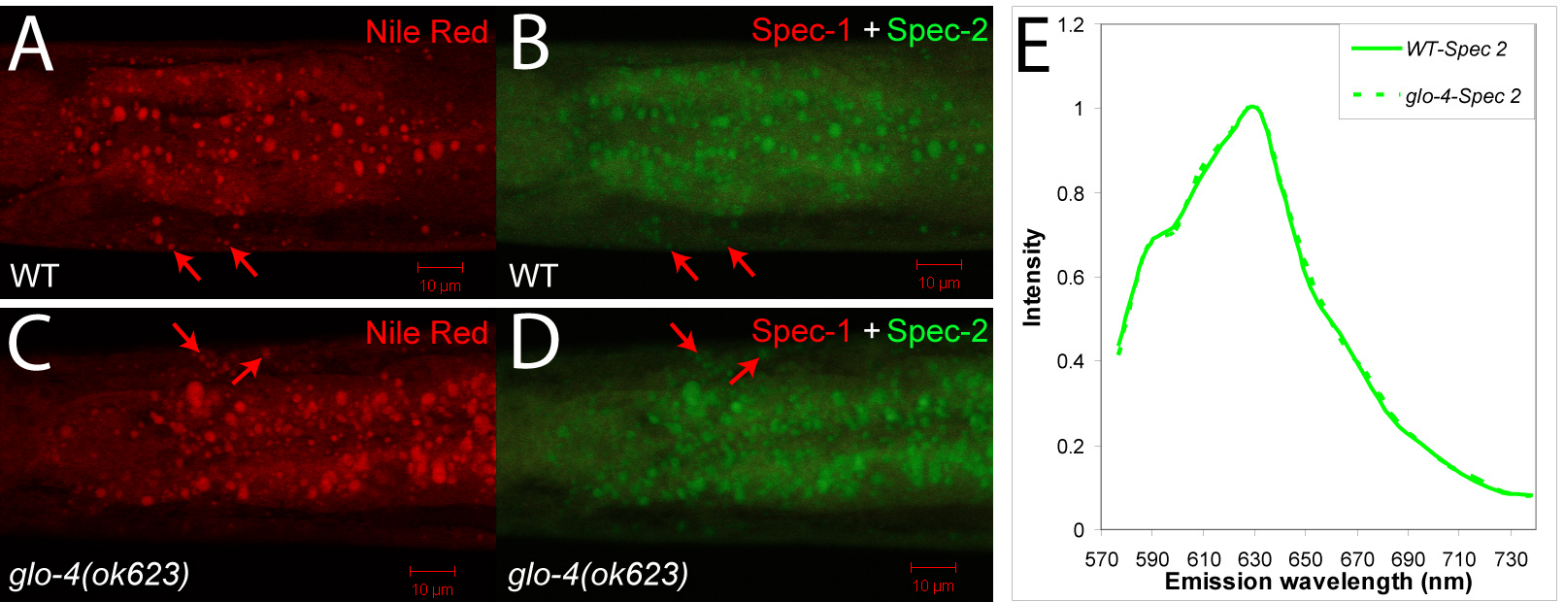
**Post-fix Nile Red staining labels lipid droplet-specific Spec-2 structures but not LRO-specific Spec-1 structures**. (A) Nile Red labelled structures in fixed wild-type animals as revealed by channel mode confocal images. (B) These labelled structures displayed lipid droplet-specific Spec-2 but not Spec-1 spectrum as revealed by linear un-mixing lambda mode confocal images. (C) Nile Red labelled structures in fixed *glo-4 *animals as revealed by channel mode confocal images. (D) These labelled structures displayed lipid droplet-specific Spec-2 but not Spec-1 spectrum as revealed by linear un-mixing lambda mode confocal images. Arrows point to structures stained by Nile Red in hypodermal cells. E. Emission spectrum profiles of Spec-2s in post-fixed wild-type and *glo-4 *animals. All images were single confocal slices. Scale bars, 10 μm.

We hypothesized that the invasive manner in which Nile Red and Oil-Red-O were delivered allowed targeting of these dyes to lipid droplets that bypassed LROs. To test this hypothesis, we stained fixed wild-type and *glo-4 *animals with Nile Red. Consistent with a previous report [20], post-fix Nile Red staining labelled spherical structures in both wild-type and *glo-4 *animals (Figure 4A and 4C), which were not otherwise vital-labelled by Nile Red in *glo-4 *mutants (Figure 1B). More interestingly, spectral analyses of emission spectra of Nile Red in wild-type and *glo-4 *animals revealed that all labelled structures displayed the same Spec-2 property as that of lipid droplets in peroxisome mutants (Figure 4B, 4D and 4E). Moreover, post-fix Nile Red staining did not label any Spec-1 structures, i.e., LROs (Figure 4B and 4D), which were otherwise the only structures labelled by Nile Red in live wild-type animals (Figure 3B).

### Lipid droplets are ubiquitous fat storage organelles with a phospholipid monolayer membrane in *C. elegans*

The above imaging and biochemical isolation experiments indicate that fat storage structures in wild-type and *glo-4 *animals possess the same properties as enlarged lipid droplets in mutants defective in peroxisomal function. Next, we sought to confirm that they also shared the same properties at the ultrastructural level, similar to mammalian lipid droplets that are characterized as electron-translucent structures with a phospholipid monolayer membrane [3].

**Figure 5:**
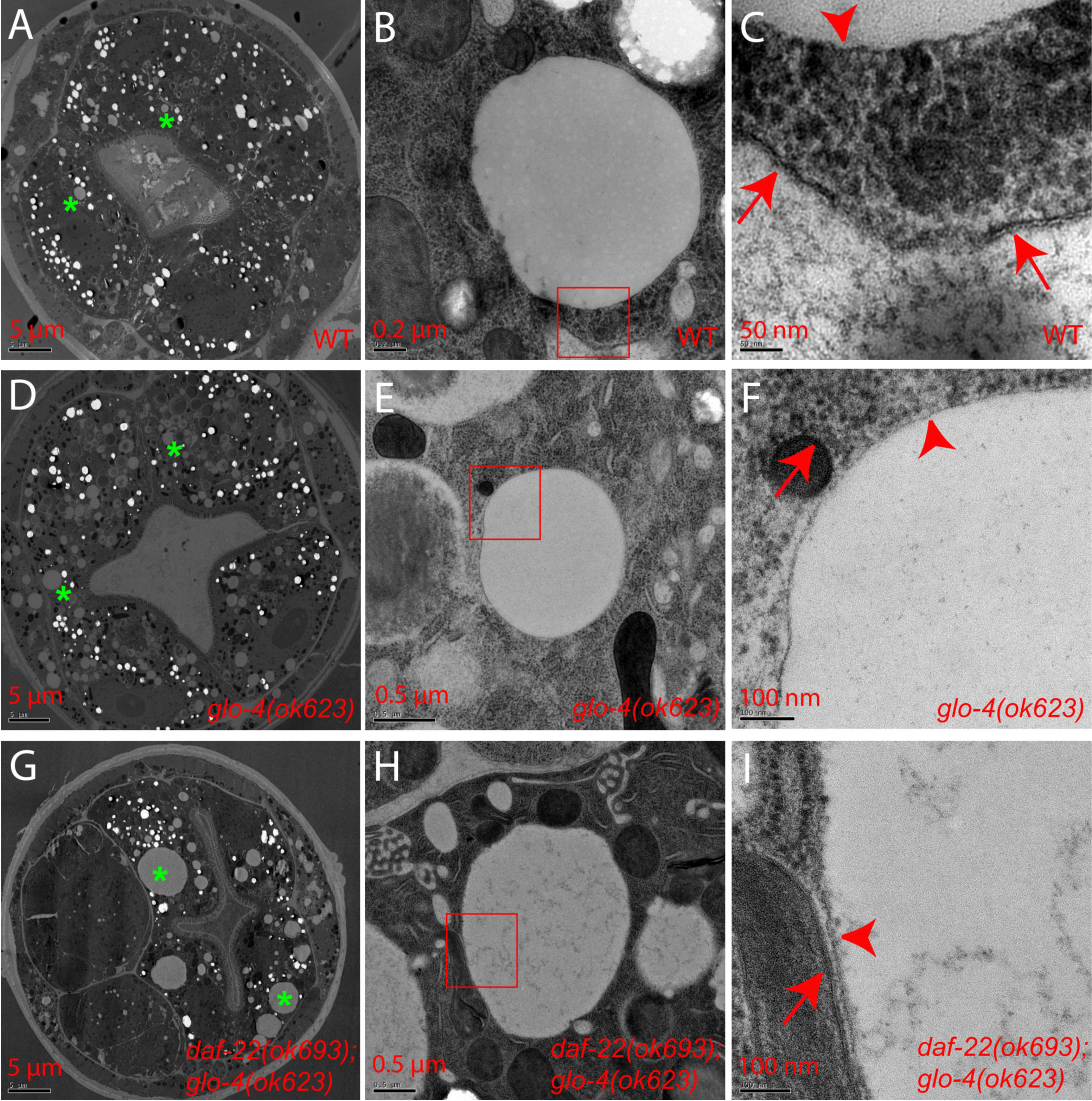
**Lipid droplets of *C. elegans *are characterized by a phospholipid monolayer membrane**. (A) Lipid droplets in wild-type animals were electron-translucent structures (asterisks). (B and C) A typical lipid droplet (boxed area) was shown to be delimited by a phospholipid monolayer membrane (arrowhead) as opposed to a phospholipid bilayer membrane (arrows) of a nearby organelle. (D-F) Lipid droplets were not eliminated by *glo-4 *mutation. (G-I) Lipid droplets in *daf-22 *mutants were not eliminated by *glo-4 *mutation.

Close inspection of electron-translucent structures in wild-type animals revealed that they were indeed similar to enlarged lipid droplets in *daf-22 *mutant animals [21]. These structures, with diameters normally smaller than 3 μm, possessed a phospholipid monolayer membrane (Figure 5A-5C), the hallmark of lipid droplets. In *glo-4 *mutants, lipid droplets were not eliminated (Figure 5D-5F). In *daf-22;glo-4 *double mutants, neither small nor large lipid droplets were eliminated (Figure 5G-5H). These results demonstrate that lipid droplets are universal fat storage structures in *C. elegans *and that lipid droplets are fundamentally distinct from LROs in their structural properties and their sensitivity to different genetic regulatory mechanisms.

## Discussion

The present study serves two purposes in the research of *C. elegans *fat storage. The first is to ascertain the organelle nature of fat storage structures. The second is to carefully compare the different vital and post-fix staining methods that aimed to label fat storage structures.

This work is based on our previous study in which we demonstrated that the enlarged spherical structures found in peroxisomal β-oxidation mutants were lipid droplets [21]. It was unclear at that time whether lipid droplets formed only when peroxisomal function was attenuated in *C. elegans*. In this paper, we provided three lines of evidence, namely, electron microscopy, biochemical isolation, vital and post-fix lipophilic dye staining and fluorescence imaging, to demonstrate that lipid droplets are ubiquitous fat storage organelles in *C. elegans*. First, we showed that in wild-type *C. elegans*, at an ultrastructural level, lipid droplets were uniformly electron-translucent structures delimited by a phospholipid monolayer membrane. This ultrastructural characteristic was previously documented for large lipid droplets in peroxisomal β-oxidation mutants in *C. elegans *[21], lipid droplets in the budding yeast [25], and lipid droplets in mammalian cells [3,26]. Such characteristic is regarded as a hallmark of lipid droplets [1,2]. Second, since lipid droplets are loaded with neutral fat such as TAG and CE, their density is much lower than water and most other intracellular organelles. This physical property allows density centrifugation-based isolation of lipid droplets from yeast and mammalian cells [3,22,23,25]. We developed a similar approach and isolated lipid droplets from wild-type and peroxisomal β-oxidation mutant *C. elegans*. And we found that the isolated lipid droplets were labelled by BODIPY. Third, we confirmed previous findings that LROs were the major organelles labelled by BODIPY and Nile Red in live animals. Nevertheless, we found that Nile Red labelled lipid droplets in live peroxisomal β-oxidation mutants, and its fluorescence emission spectrum was distinct from that of Nile Red in LROs. Nile Red emission spectrum shift was previously documented as neutral fat-dependent [27]. The emission spectrum shift was also found to be specific to lipid droplets in mammalian macrophages [28].

**Figure 6:**
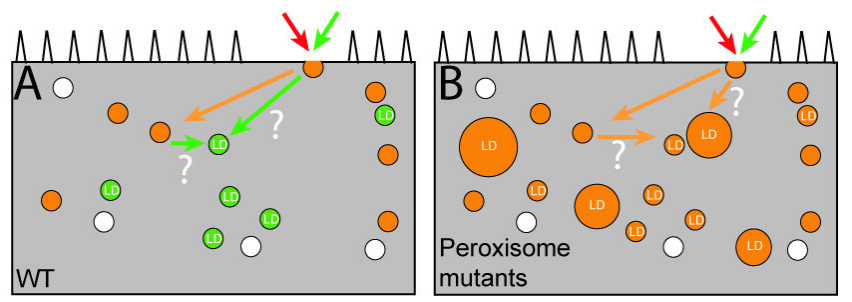
**A model of BODIPY and Nile Red trafficking in live wild-type animals and peroxisomal β-oxidation mutants**. (A) A schematic diagram of possible BODIPY (green) and Nile Red (red) trafficking in gut epithelial cells in wild-type animals. A single gut epithelial cell is diagrammed as a rectangular box with microvilli (triangles) pointing to the gut lumen. Gut epithelial cells are rich in vesicular structures, which include LROs (orange vesicles), lipid droplets (green vesicles labelled as LD), and other vesiclular organelles (white). Both BODIPY and Nile Red are endocytosed into the gut epithelial cell and trafficked through endolysosomal pathways to LROs (orange arrow and orange vesicles). Hypothetically, BODIPY can be targeted to lipid droplets from LROs and or from endosomes (green arrows question marked). (B) In peroxisomal β-oxidation mutants, lipid droplet size increases (large orange vesicles). Nile Red as well as BODIPY can be targeted to lipid droplets (orange arrows question marked).

Our identification of lipid droplets as the fat storage organelles in *C. elegans *has important implications for the principles and validity of previous fat staining methods. As shown in this study and several previous studies, Nile Red and BODIPY label LROs in live wild-type *C. elegans *[7,8,19,21]. In addition, we showed that BODIPY but not Nile Red can label lipid droplets in wild-type *C. elegans*. Since the absence of LROs in *glo *mutants does not alter TAG levels [19], LROs are unlikely to be the sites of fat storage. Thus, fluorescence intensities of BODIPY or Nile Red vital staining cannot be used as a sole indicator of fat levels. This view agrees with those of two previous reports [8,20]. Why does BODIPY label both LROs and lipid droplets and why does Nile Red label only LROs in wild-type *C. elegans*? Vital dyes are applied onto the *E. coli *lawn and ingested by *C. elegans *into the gut lumen. It is plausible that the vital dyes are endocytosed, recycled, and delivered to lysosomes for degradation in gut epithelial cells [7,29-31]. BODIPY is different from Nile Red in that it is conjugated to a fatty acid moiety. In mammalian cells, fatty acids and fatty acid moieties are released from lysosomes and targeted to lipid droplets [32]. It appears that a similar process happens for BODIPY fatty acid analogs but not for Nile Red in wild-type *C. elegans *(Figure 6A). Why does Nile Red as well as BODIPY label lipid droplets besides LROs when peroxisomal β-oxidation is impaired? The exact mechanism is currently unknown. The peroxisomal β-oxidation pathway and endolysosomal pathway genetically interact to affect larval development and body length (unpublished data). Similar genetic interactions may underlie the trafficking of Nile Red from endosomes and/or lysosomes to lipid droplets (Figure 6B). Physical contact between peroxisomes and lipid droplets may also be involved [33]. Why do the two post-fix methods reveal fat levels more closely [8,20]? The post-fix labelling methods involve permeating cell membrane and intracellular membrane invasively by isopropanol and/or freeze/thaw treatments [8,20]. The delivery of Nile Red and Oil-Red-O to lipid droplets thus bypasses the endolysosomal pathway and LROs. The post-fix staining procedure may also compromise the integrity of LROs and make them un-stainable.

The present study should allow re-interpretation of previous studies in *C. elegans *fat research and guide the design of future qualitative and quantitative studies of lipid droplets. For example, we showed that BODIPY could label lipid droplets with low intensity in live wild-type animals while Nile Red and BODIPY could label both large and small lipid droplets in live peroxisomal β-oxidation mutant animals. Our results highlight the complexity of intracellular trafficking of lipophilic dyes which should be examined carefully. On one hand, it suggests that mutants showing changes in vital Nile Red and BODIPY fluorescence intensity may actually have alterations in lysosome/LRO proliferation or function. On the other hand, it lends support to the use of these two vital dyes for lipid droplet labelling in peroxisomal mutant animals and animals of other yet unexplored genetic backgrounds, provided that independent methods are available to distinguish lipid droplets from LROs. Because of the dual labelling by BODIPY in our peroxisomal β-oxidation mutants, we previously opted for GC-MS to gauge fat levels and we measured volumes instead of fluorescence intensities to study lipid droplets larger than 3 μm in diameter [21]. The spectral imaging of Nile Red and the post-fix Nile Red staining should now allow measurement of volumes of lipid droplets of all sizes. We also anticipate that, before their acceptance as alternative live imaging techniques for fat levels in *C. elegans*, label-free imaging approaches based on Raman scattering [11] should be subjected to rigorous analyses using a combination of genetic manipulation of lipid droplets and LROs, biochemical isolation, spectral analysis, electron microscopy, and lipid analytical chemistry.

## Conclusions

In conclusion, we have ascertained lipid droplets as the ubiquitous fat storage organelles in *C. elegans*. Our results demonstrate the strengths and weaknesses of different dye labelling methodologies for lipid droplets in *C. elegans*, which will aid future experimental design in this powerful genetic system.

## Methods

### Strains and Transgenes

The wild-type strain was N2 Bristol. The following mutant and transgenic strains were used: *daf-22(ok693)*, *glo-4(ok623)*, *dhs-28(hj8)*, *daf-22(ok693);glo-4(ok623)*, *Is[ges-1p::glo-1::gfp](hjIs9)*, *dhs-28(hj8);Is[ges-1p::glo-1::gfp](hjIs9)*, *glo-4(ok623);Is[ges-1p::glo-1::gfp](hjIs9)*.

### Bacteria and Nematode Growth Medium (NGM) Plates

Bacteria culture and seeding of 6-cm and 10-cm NGM plates were the same as previously described [21].

### BODIPY and Nile Red Vital Staining

BODIPY and Nile Red vital staining in live animals was essentially the same as previously described [21]. 100 μL 5 μM green BODIPY (#D-3823, Invitrogen) or red BODIPY (#D-3835, Invitrogen) in 1X phosphate buffer saline (PBS, pH 7.2) was added onto the ~2 cm-in-diameter OP50 bacteria lawn of a 6-cm NGM plate. To vital-label animals in large quantity, 1 mL 12.5 μM green or red BODIPY was added onto the 10 cm-in-diameter OP50 bacteria lawn of a 10-cm NGM plate. The plates were immediately dried in laminar flow hood and were used to grow and stain animals. For Nile Red staining, 100 μL 5 μg/mL Nile Red (#N-1142, Invitrogen) in PBS was added onto the ~2 cm-in-diameter OP50 bacteria lawn of a 6-cm NGM plate. 1 mL 12.5 μg/mL Nile Red in PBS was added onto the 10 cm-in-diameter OP50 bacteria lawn of a 10-cm NGM plate. The Nile Red plates were immediately dried in laminar flow hood and were equilibrated at room temperature for 24 hr before use.

### Confocal Microscopy and Spectral Analysis

Channel mode confocal images of BODIPY, Nile Red, and GFP fluorescence in late stage L4 animals were acquired essentially the same as previously described [21]. To compare BODIPY and Nile Red fluorescence of wild-type, *glo-4*, and *daf-22;glo-4 *animals, we used imaging parameters that detected fluorescence of wild-type animals within the linear range. In order to carry out emission spectrum analyses of vital Nile Red fluorescence, lambda mode confocal imaging and image processing by linear un-mixing were conducted [34]. A Zeiss LSM 510 inverted confocal microscope equipped with a META detector was used. Images were acquired with a C-Apochromat 40X/1.20 W Korr UV-VIS-IR M27 objective. Key parameters were: excitation laser DPSS 561 nm, 15 mW, 15%; emission spectrum 577-738 nm at 10 nm per channel; pinhole, 90 μm; optical zoom, 2X; line scan, mean of 4; gain and offset were tailored to individual images so that no pixel was saturated in each channel. Lambda mode images were acquired for non-stained wild-type and *dhs-28 *animals as negative controls. No autofluorescence was detected. Two types of spectra were identified for Nile Red-stained structures in *dhs-28 *intestinal cells in lambda mode images. The spectrum with peak shifted towards a shorter wavelength was classified as "Spec-2", while the other, "Spec-1". In wild-type animals, only Spec-1 was identified. The two spectral profiles of *dhs-28 *animals were used to linearly un-mix lambda mode images of wild-type, *dhs-28*, *dhs-28;Is[ges-1p::glo-1::gfp]*, and *daf-22;glo-4 *animals with the same parameter settings. Spec-1s were pseudo-colored red. Spec-2s, green. No apparent co-localization of red and green structures and minimal un-assigned pixels indicated reliable spectral classification and un-mixing. Lambda imaging of red BODIPY fluorescence in *dhs-28;Is[ges-1p::glo-1::gfp] *animals and in wild-type animals were essentially the same as that of Nile Red fluorescence except that the emission spectrum spanned 566-738 nm at 10 nm per channel. Only one emission spectrum was identified in Red BODIPY-stained *dhs-28 *and wild-type animals. For each imaging experiment, 10-30 animals were imaged.

### Post-fix Nile Red Staining

Late L4 stage wild-type and *glo-4 *animals were fixed and stained essentially as previously described [20]. In brief, late L4 animals were fixed in 1% formaldehyde/PBS for 10 minutes. Then samples were immediately frozen on dry ice/ethanol and thawed on running tap water. Samples were frozen and thawed again, 3 times in total. Samples were washed 3 times with 1X PBS, dehydrated in 60% isopropanol for 2 minutes, stained with 0.5 mL 1 μg/mL Nile Red in 60% isopropanol for 30 minutes. Stained samples were then washed 3 times with 1X PBS, re-hydrated in 1X PBS, and mounted in 1X PBS onto agarose-padded slides for fluorescence imaging. Channel mode imaging and lambda imaging were conducted the same as described in previous section.

### Isolation of Lipid Droplets

To isolate BODIPY-labelled lipid droplets, 4,000 freshly hatched wild-type or *dhs-28 *animals were loaded onto one 10-cm NGM/OP50 plate supplemented with green or red BODIPY. Animals were allowed to grow to 1-day-adult stage, i.e., 24 hr past L4 stage. Animals were harvested and washed 4 times with 1X PBS/0.001% Triton X-100. Worm pellet was re-suspended in 1X PBS added with protease inhibitors on ice. Worm samples were then homogenized by 20 strokes using a dounce homogenizer with a tight-fit pestle. Homogenates were transferred in 10 mL 1X PBS/protease inhibitors into a falcon tube. Homogenates were centrifuged for 10 minutes at 1000 g at 4°C. 1 mL top layer was aspirated and transferred into a new tube and washed by adding 9 mL 1X PBS/protease inhibitors. The tube was centrifuged in the same way. The 1 mL top layer was collected and washed with 9 mL 1X PBS/protease inhibitors, and centrifuged again. The final 1 mL top layer was collected for further analysis. To image isolated lipid droplets, the final top layer fraction was mounted onto a cytometer and imaged on a Zeiss Observer.Z1 inverted microscope with a 40X/NA1.20 W objective. Epi-fluorescence imaging parameters were set so that the BODIPY fluorescence of lipid droplets isolated from *dhs-28 *animals was detected within the linear range. Lipid droplets from wild-type animals were imaged with the same parameters.

To isolate Nile Red-labelled lipid droplets from *dhs-28 *animals, 4,000 freshly hatched L1s were loaded onto each of three 10-cm NGM/OP50/Nile Red plates. The 12,000 animals were allowed to grow to late stage L4 and were then harvested. Lipid droplets were isolated in the same way as for BODIPY-labelled animals. The post-nuclear cytoplasmic fraction generated during the first centrifugation step was further spun at 100,000 g for 30 min at 4°C. The membrane pellet was re-suspended in 1 mL 1X PBS/protease inhibitors. Lipid droplet fraction and cytoplasmic membrane pellet fraction were mounted onto a cytometer for confocal imaging. Lambda mode confocal imaging was conducted for the two fractions in the same way as described in "Confocal Microscopy and Spectral Analysis" section. Experiments were repeated three times. More than 30 lipid droplets and 30 membrane pellets were imaged.

To prepare lipid droplet fraction and cytoplasm fraction for western blot experiment, 4000 freshly hatched *dhs-28;Is[ges-1p::glo-1::gfp] *L1s were loaded onto a 10-cm NGM/OP50 plate. Animals were harvested at late L4 stage. One plate of animals was treated as one sample. The sample was subjected to lipid droplet isolation with the same procedure, except that the extraction volume was scaled down from 10 mL to 1 mL and the lipid droplet fraction volume was scaled down from 1 mL to 0.1 mL. The 0.1 mL final lipid droplet fraction and the 0.9 mL post-nuclear cytoplasm fraction generated during the first centrifugation step were concentrated and extracted for protein in an equal final volume of 60 μL. 7.5 μL of each protein sample was loaded onto a SDS-PAGE gel and was subjected to western blot using a custom made rabbit anti-GFP antibody (Y2769). Wild-type lipid droplet fraction and cytoplasm fraction were prepared in the same way and were loaded alongside as a negative control. Experiments were conducted two times. Three samples each time.

### Electron Microscopy

Electron microscopy was conducted the same as previously described [21].

## Authors' contributions

SOZ and HYM designed research; SOZ, RT and FG performed research; SOZ and HYM wrote the paper. All authors read and approved the final version of the manuscript.

## Supplementary Material

Additional file 1 **Dual labelling of BODIPY in live animals**. In live wild-type animals, red BODIPY labelled GLO-1::GFP-encircled LROs with high intensity (arrowheads). It also labelled non-GLO-1::GFP-encircled structures with low intensity (arrows). Images were single confocal slices. Click here for file

Additional file 2 **Isolated lipid droplets free of LRO marker protein GLO-1::GFP**. Lipid droplet fraction and cytoplasm fraction were prepared from stage L4 *dhs-28;glo-1::gfp *animals. Triplicates. Fractions were probed for the presence of GLO-1::GFP protein using a GFP antibody. GLO-1::GFP (predicted MW, ~50 kD, arrow) was detected in cytoplasm fractions but not in lipid droplet fractions. Asterisk denotes a non-specific protein recognized by the GFP antibody that is present in wild-type control (WT). Click here for file

Additional file 3 **Distinct emission spectra of lipid droplets and membrane pellets isolated from *dhs-28 *animals that were vital-labelled by Nile Red**. (A) In stage L4 *dhs-28 *animals that were grown and stained on 10-cm NGM/OP50/Nile Red plates, LROs displayed Spec-1 emission spectrum (pseudo-colored in red) and lipid droplets displayed Spec-2 (pseudo-colored in green). Similarly, membrane pellets (B) and lipid droplets (C) isolated biochemically from these *dhs-28 *animals displayed Spec-1 and Spec-2 respectively. (D) Emission spectrum profiles of Spec-1s (red line) and Spec-2s (green line) of intact *dhs-28 *animals and Spec-1s (dashed red line) and Spec-2s (dashed green line) of isolated membrane pellets and lipid droplets. Intensity values were normalized. Click here for file

Additional file 4 **The same fluorescence emission spectrum of BODIPY-labelled LROs and lipid droplets**. In live *dhs-28 *mutants, red BODIPY labelled both lipid droplets and LROs (marked by GLO-1::GFP). However, red BODIPY displayed the same emission spectrum (colored in gold) in lipid droplets and LROs. LD, lipid droplet. Images were single confocal slices. Click here for file
