# Supplementary figures and images for: Lipid droplets as ubiquitous fat storage organelles in C. elegans
Source: BMC Cell Biol. 2010 Dec 8;11:96. doi: 10.1186/1471-2121-11-96 (PMC3004847; doi:10.1186/1471-2121-11-96)

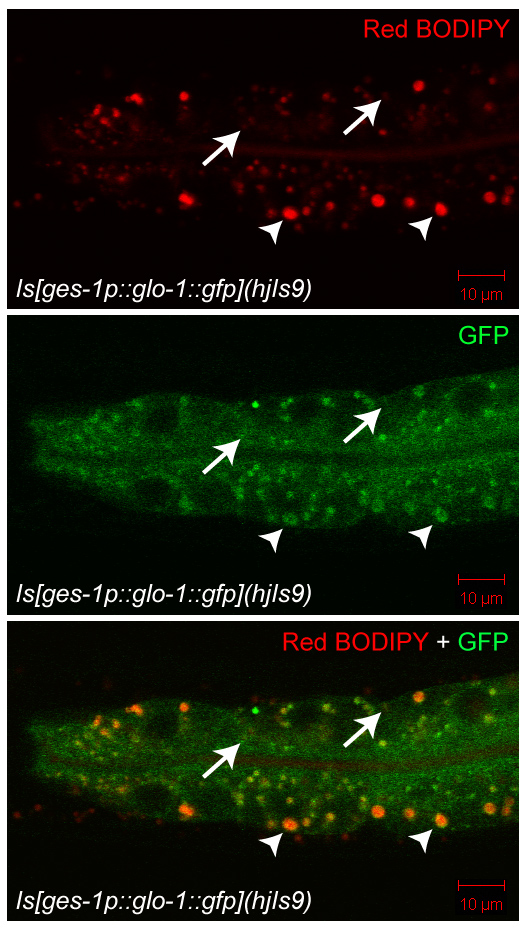

Supplement: Additional file 1 — Dual labelling of BODIPY in live animals. In live wild-type animals, red BODIPY labelled GLO-1::GFP-encircled LROs with high intensity (arrowheads). It also labelled non-GLO-1::GFP-encircled structures with low intensity (arrows). Images were single confocal slices. [file 1471-2121-11-96-S1.JPEG]

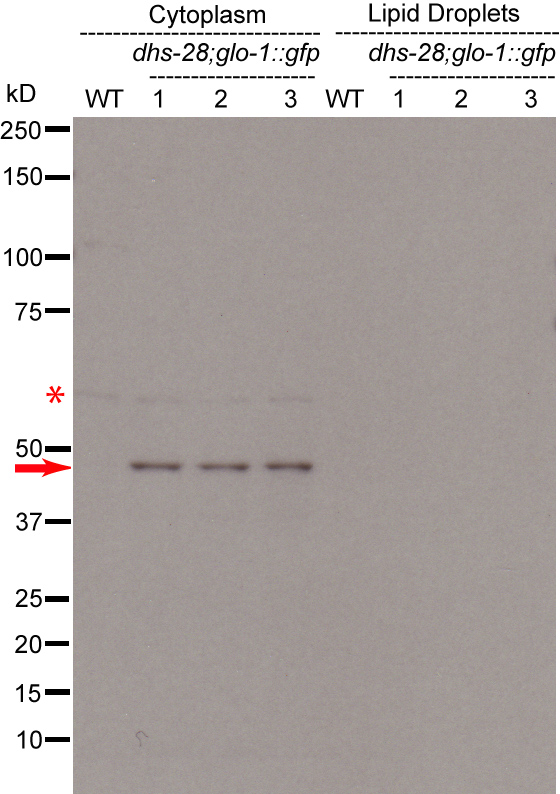

Supplement: Additional file 2 — Isolated lipid droplets free of LRO marker protein GLO-1::GFP. Lipid droplet fraction and cytoplasm fraction were prepared from stage L4 dhs-28;glo-1::gfp animals. Triplicates. Fractions were probed for the presence of GLO-1::GFP protein using a GFP antibody. GLO-1::GFP (predicted MW, ~50 kD, arrow) was detected in cytoplasm fractions but not in lipid droplet fractions. Asterisk denotes a non-specific protein recognized by the GFP antibody that is present in wild-type control (WT). [file 1471-2121-11-96-S2.JPEG]

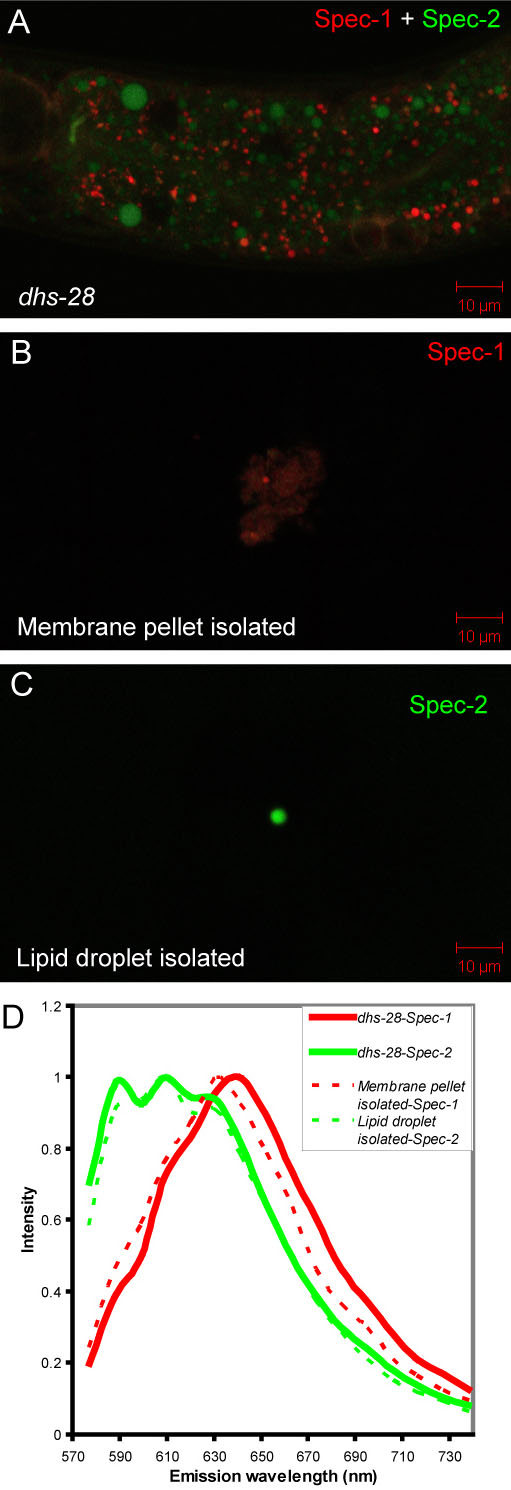

Supplement: Additional file 3 — Distinct emission spectra of lipid droplets and membrane pellets isolated from dhs-28 animals that were vital-labelled by Nile Red. (A) In stage L4 dhs-28 animals that were grown and stained on 10-cm NGM/OP50/Nile Red plates, LROs displayed Spec-1 emission spectrum (pseudo-colored in red) and lipid droplets displayed Spec-2 (pseudo-colored in green). Similarly, membrane pellets (B) and lipid droplets (C) isolated biochemically from these dhs-28 animals displayed Spec-1 and Spec-2 respectively. (D) Emission spectrum profiles of Spec-1s (red line) and Spec-2s (green line) of intact dhs-28 animals and Spec-1s (dashed red line) and Spec-2s (dashed green line) of isolated membrane pellets and lipid droplets. Intensity values were normalized. [file 1471-2121-11-96-S3.JPEG]

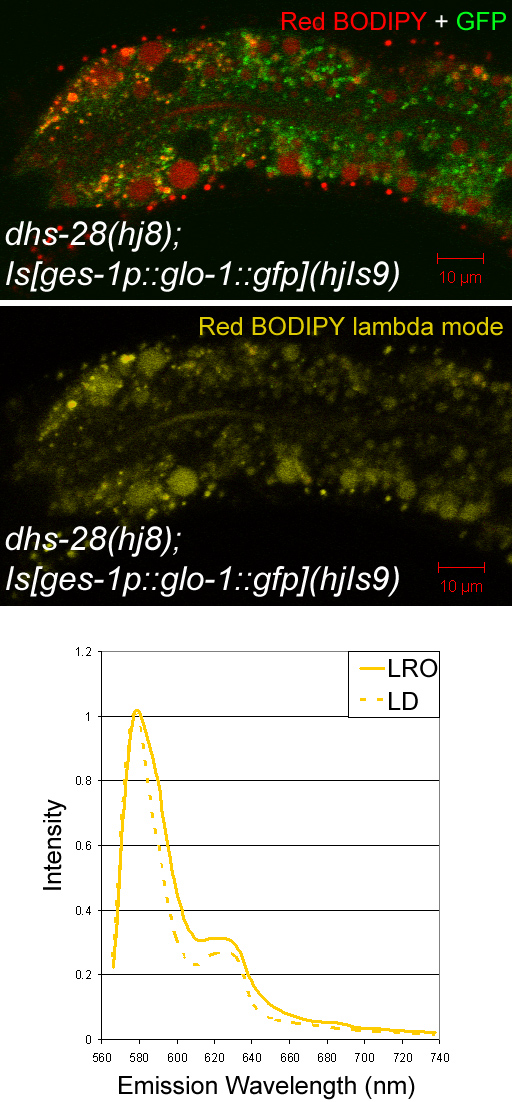

Supplement: Additional file 4 — The same fluorescence emission spectrum of BODIPY-labelled LROs and lipid droplets. In live dhs-28 mutants, red BODIPY labelled both lipid droplets and LROs (marked by GLO-1::GFP). However, red BODIPY displayed the same emission spectrum (colored in gold) in lipid droplets and LROs. LD, lipid droplet. Images were single confocal slices. [file 1471-2121-11-96-S4.JPEG]
